# Molecular Epidemiology and Pathogenic Characterization of Novel Chicken Infectious Anemia Viruses in Henan Province of China

**DOI:** 10.3389/fvets.2022.871826

**Published:** 2022-03-28

**Authors:** Xin-Wei Wang, Jie Feng, Jia-Xin Jin, Xiao-Jing Zhu, Ai-Jun Sun, Hua-Yuan Liu, Jing-Jing Wang, Rui Wang, Xia Yang, Lu Chen, Yi-Fei Liao, Guo-Qing Zhuang

**Affiliations:** ^1^College of Veterinary Medicine, Henan Agricultural University, Zhengzhou, China; ^2^Wolong Animal's Sanitation Administration, Nanyang, China; ^3^Division of Infectious Disease, Department of Medicine, Brigham and Women's Hospital and Harvard Medical School, Boston, MA, United States

**Keywords:** epidemiology, immunosuppressive disease, live attenuated vaccine, recombination, chicken infectious anemia

## Abstract

Chicken infectious anemia (CIA) is an immunosuppressive disease caused by the chicken infectious anemia virus (CIAV) resulting in heavy economic losses once an outbreak is established. This study conducted a systematic analysis of the epidemiology and pathology of CIA in Henan province, China. A total of 437 clinical tissue samples and 120 poultry disease-related live attenuated vaccines were collected during 2017–2020; of which 45 were positive for CIAV nucleic acid, with a positive rate of 8.08%. Our results showed that genome sequence similarity among a total of 12 CIAV isolates was high, and ranged from 97.1 to 99.3%, and their similarity to the vaccine strains Cux-1 and Del-Ros ranged from 97.8 to 98.6%. However, There were mutations in the locus of the major capsid proteins VP1, VP2, and VP3 among all isolates. The subsequent sequence analysis indicated that the isolates of HN-4 and HN-8 showed genetic recombination and follow up animal experiments revealed that HN-4 might be a pathogenic strain. Our results reveal that both field infection and non-CIAV vaccines contamination promote the epidemiology of CIAV in China and some dominant epidemic viruses have undergone recombination and evolution. This study provides important information to help with the prevention and control of CIAV in the poultry industry.

## Introduction

Chicken infectious anemia (CIA) is a viral disease caused by the chicken infectious anemia virus (CIAV). The disease is characterized by aplastic anemia and lymphatic atrophy (1). CIAV is a non-encapsulated, symmetrical icosahedral virus particle, with an average diameter of 25 nm. The genome of CIAV is single-stranded, circular, negative-stranded covalently closed DNA, of ~2 kb, which contains three partially overlapping open reading frames (ORF) encoding VP1, VP2, and VP3, respectively (2). VP1 represents the nucleocapsid protein of CIAV and is the main immunogen, which encodes an arginine-rich polypeptide with a molecular weight of approximately 50 Ka (3). VP2 is an auxiliary scaffold protein required for CIAV assembly, which helps VP1 to form the correct conformation (4), and both VP1 and VP2 can cooperate with each other to induce an immune response. VP3 represents a functional protein of the virus, which is also known as apoptin (5) and studies have found that transfection of the *VP3* gene into chicken monocyte cultures, can induce apoptosis, highlighting its importance in CIAV infection (6).

CIAV mainly invades the bone marrow and thymus of the central immune organ, leading to yellowing of the bone marrow and atrophy of the thymus, and can also cause aplastic anemia and immunosuppression (7). As one of the important poultry diseases, CIAV infection can either directly reduce the protective efficacy of certain vaccines, or enhance pathogenicity of attenuated vaccines, leading to immune failure (8, 9). The natural mortality rates from CIAV infection in the clinic is approximately 10–20%. Due to the low mortality rates and masked infectivity, CIAV has often been neglected in clinical settings (10). However, the latent infection causes chicken immunosuppression, which enhances the invasion of other opportunist pathogens including bacteria and viruses, which ultimately lead to chicken growth and development disorders (11). Significantly, CIAV can transmit both horizontally and vertically. Horizontal transmission occurs in the flock usually from the feces, danders or feathers, mainly through oral infection. More importantly however, vertical infection is derived from breeding eggs, which can easily result in an outbreak of CIA in the offspring. Furthermore, previous studies have highlighted the fact that vertical transmission still exists, even if there is an immune response in infected hens (12).

CIAV infection has recently spread globally causing severe economic losses. For example, 32 CIAV sequences were identified in 2010 in South Korea (13). In India, 351 serum samples were positive out of 404 (86.88%) collected from chicken flocks in 11 poultry farms (14), and in northern Vietnam, there were 74 positive tests from 119 samples (62.2%) collected from 64 farms (15). In China, CIAV has been detected in poultry farms from most provinces, and has been successfully isolated both from live non-CIAV vaccines and specific pathogen free (SPF) chickens (16), which may explain the reason for the CIAV contamination found in the field. Interestingly, co-infection of four immunosuppressive pathogens including CIAV has been detected in Shandong Province (17, 18). Recently, 1,187 clinical samples from major poultry farms nationwide from 2017 to 2020, have been tested for co-infection with six immunosuppressive pathogens including CIAV (19). These surveys have confirmed the widespread nature of CIAV in China, which has seriously affected the development of the poultry industry. However, there is a paucity of information relating to CIAV infection in the central region of China. Therefore, an epidemiological investigation of CIA was carried out on samples collected from chicken farms and live attenuated non-CIAV vaccines in the markets of Henan Province, and the genomes of CIAV epidemic strains in this region were systematically analyzed. Furthermore, the pathogenesis of one recombinant strain has been investigated further.

## Materials and Methods

### Sample Collection

During 2017–2020, the spleen samples were collected from 5–10 weeks old sick chicken of 437 flocks produced throughout Henan province, China, diagnosed by the Poultry Disease Research Institute of Henan Agricultural University. In brief, spleens from suspected chickens containing CIAV were cut into small pieces and place into a sterile tube. Samples were then homogenized three times in phosphate buffer saline (PBS) using small steel balls in an automated tissue grinder. Next, the samples were freeze/thawed three times to encourage the release of the virus. The viral supernatant was isolated by centrifugation at 8,000 rpm and then transferred into a new sterile tube. A total of 120 non-CIAV live attenuated vaccines were collected from 24 manufacturers and five different batches for CIAV detection.

### Virus Amplification

Marek's disease lymphoblastoid cells (MSB1), a kind gift from Professor Jun Ji, Nanyang Normal University, Henan province were cultured in 1640 culture medium with 10% fetal bovine serum (Solarbio, Beijing, China). When the cells reached confluency at 5 × 10^5^ cells/cm^3^, they were centrifuged at 1000 rpm for 5 min and washed twice with sterile PBS to remove residual serum. Then the CIAV containing supernatant was added for virus adsorption for 1 h. After this step, MSB1 cells were cultured in 5 mL of 1640 medium containing 2% fetal bovine serum and when visible enlarged and broken CIAV infected cells could be seen, the infected cells were passaged blind five times with fresh MSB1 cells, for viral amplification.

### Nucleic Acid Extraction and CIAV Detection

Genomic DNA was extracted from the chicken samples using a commercial kit (TIANamp Genomic DNA Kit, Tiangen, Beijing, China) and used according to the manufacturer's instructions. Three pairs of primers for amplification of the CIAV genome were designed and synthesized by Primer Premier 5.0 (Sangon Biotech, Shanghai, China). The primers for gene amplification were designed based on CUX-1 sequence (accession number M55918). The first primer pair were as follows: forward primer 5′-GCATTCCGAGTGGTTACTATTCC-3′, reverse primer 5′-CGTCTTGCCATCTTACAGTCTTAT-3′; and these primers were expected to produce a predicted amplicon size of 842 bp. The second primer pair were as follows: forward primer 5′-CGAGTACAGGGTAAGCGAGCTAAA-3′, reverse primer 5′-TGCTATTCATGCAGCGGACTT-3′; and these primers produced a predicted amplicon size of 990 bp. The final set of primers were as follows: forward primer 5′-GAAAATGAGACCCGACGAGCAACAG-3′, reverse primer 5′-GATTCGTCCATCTTGACTTTCTGTG-3′, and these primers produced a predicted amplicon size of 736 bp. There three pairs of primers are used for CIAV detection and sequencing analysis. The polymerase chain reaction (PCR) was performed in a total volume of 50 μL, containing 18 μL of 2 x Taq Master Mix, 1 μL (10 μm/L) of each primer, 27 μL of distilled water, and 3 μL of template DNA. The PCR cycling conditions for amplification were as follows: 95°C pre-denaturation for 5 min; 95°C denaturation for 30 s, 60°C annealing for 30 s, and 72°C extension for 30 s. Amplification was performed over 35 cycles with a final extension at 72°C for 10 min. The PCR products were then resolved using 1% agarose gel electrophoresis, and included both positive and negative controls.

### Sequencing Analysis

The PCR products were purified with a PCR gel recovery kit and cloned into PMD18-T vectors and sent to Sangon Biotech for sequencing. The sequencing results were analyzed by the DNASTAR7.1 (DNASTAR, Inc., WI, USA), and then aligned using the BLAST program on the National Center for Biotechnology Information (NCBI) website (www.ncbi.nlm.nih.gov) to reference strains (Supplementary Table 1). Next, the MEGA 6.0 software (https://www.megasoftware.net) was used for phylogenetic analysis. The RDP 4.0 software was used for recombination analysis of the isolates. The positive recombinant isolate was determined by at least five methods of RDP, GENECONV, BootScan, MaxChi, Chimera, SiScan, Phyl-Pro, LARD And 3Seq (20).

### Animal Experiments

SPF chickens (Qian yuan hao biotechnology Co, Zhengzhou, China) were used for all animal experiments. Forty one-day-old SPF white broilers were wing-banded upon hatching, and housed in isolators, and randomly sorted into experimental groups. An additional group was used as control. To determine the pathogenic properties of the NH-4 isolate, experimental groups from one-day-old SPF chickens were inoculated with 0.1 ml of HN-4 in the muscle tissue, whereas the chickens in the control group were inoculated with an identical volume of physiological saline. All chickens that had died during the experiment (21 days), or were euthanized at the end of the experiment, were necropsied and examined for CIAV-related pathology.

To evaluate the effect of HN-4 on body weight and lymphoid organs, three chickens in each group were euthanatized at 7, 14, and 21 days post-infection, and their body weight and lymphoid organ weights (thymus and bursa) were measured. The ratio of the lymphoid organs was expressed as the weight of lymphoid organ divided by the body weight for each chicken, multiplied by one hundred.

For hematoxylin-eosin (H&E) staining, tissues were formalin fixed and paraffin embedded and 6–8 μm-thick sections were prepared. H&E staining was carried out following regular procedures and sections were examined under a microscope.

### Data Analysis

The ratio of the lymphoid organs and survival rates were analyzed using GraphPad Prism version 8.0.1 software (GraphPad Software, Inc. La Jolla, CA). Statistical *t*-test analysis was used for each data point, which represented a triplicate average and a value of *P* < 0.05 was considered statistically significant.

## Results

### Complete Gene Sequence Analysis of Isolated CIAVs

A total of 41 spleen samples were detected to be CIAV positive from 437 sick chickens collected from June 2017 to January 2020 in Henan province. The overall positive rate was 9.4% (95% CI: 6.8–12.5%). Four CIAV positive vaccines in 120 non-CIAV live attenuated vaccines were detected, and the overall positive rate was 3.33% (95% CI: 0.9–8.3%) (Table 1). A total of 12 CIAV strains were isolated and identified by PCR from chicken samples and as shown in Supplementary Figure 1, the amplicon sizes were 842 bp, 990 bp, and 736 bp, respectively, and this was consistent with prediction.

**Table 1:** CIAV test from collected spleens and attenuated vaccine circulating in the market.

**Sample type**	**Number of samples tested**	**Number of positive samples**	**Positive rate (%)**
Spleen	437	41	9.4
NDV + IB vaccine strain	53	3	5.66
FPV vaccine strain	31	1	3.23
NDV vaccine strain	34	0	0
MS vaccine strain	1	0	0
FPV + ILTV vaccine strain	1	0	0

*NDV, Newcastle disease virus; IB, Infectious bronchitis; FPV, Feline parvovirus; MS, Multiple sclerosis; ILTV, Infectious laryngotracheitis virus*.

The whole genome sequences of 12 isolates were amplified and aligned to the reference strain. Four strains of CIAV virus (HN-1, HN-8, HN-9, HN-12) had a similarity between 98.1 and 99.7%, of which, HN-1 and HN-9 had the highest similarity. These four isolates are more closely related to the American strain L14767.1, with a similarity of over 98%. Compared to the vaccine strains that have been widely used worldwide, the 12 isolates had high sequence similarity with the Del-Ros vaccine strain ranged from 97.8 to 98.6%, but was less than 98% with Cux-1 vaccine strain (Supplementary Figure 2).

**Figure 1:**
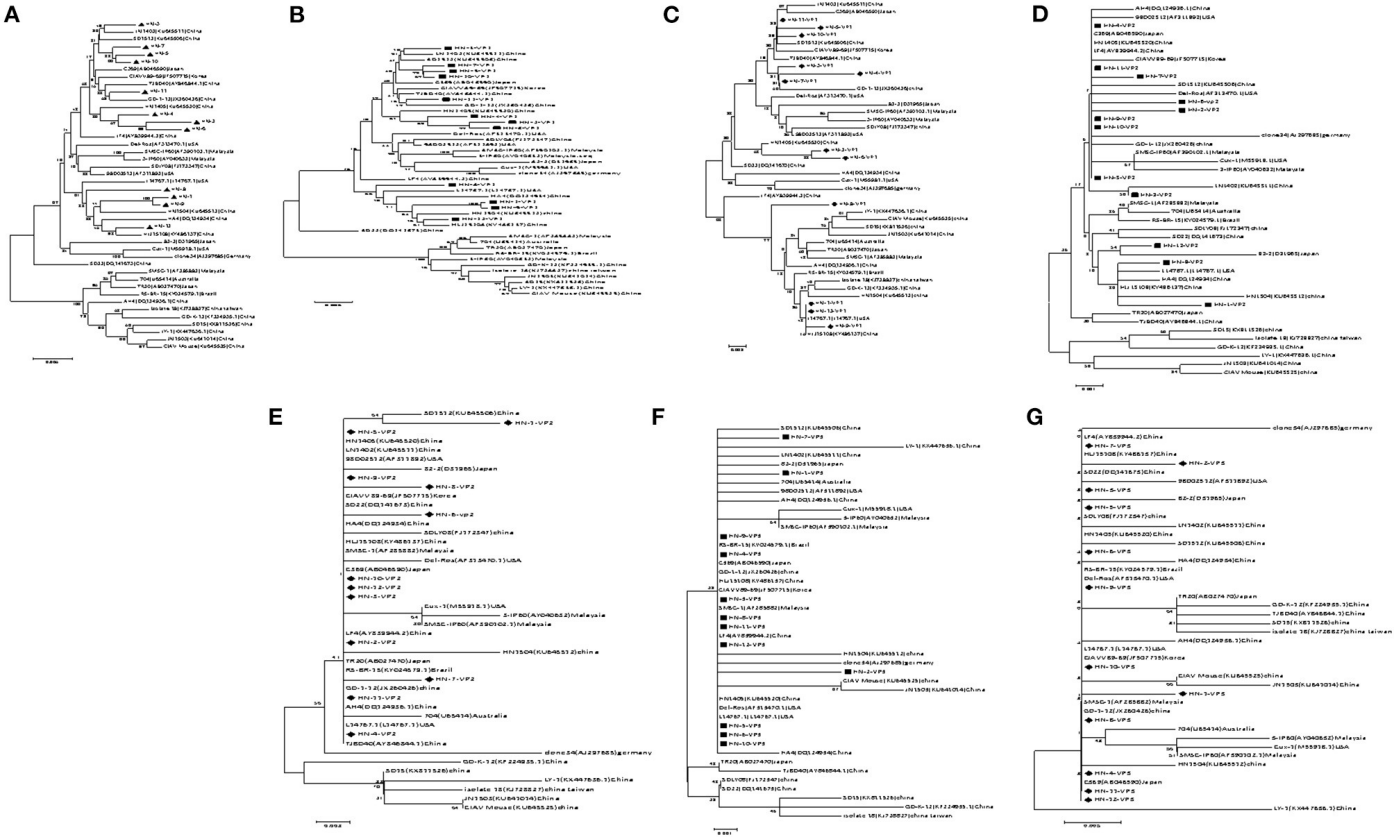
Evolutionary tree analysis of **(A)** Whole genome sequence evolutionary tree analysis; **(B)** Nucleotides of VP1; **(C)** Amino acids of VP1; **(D)** Nucleotides of VP2; **(E)** Amino acids of VP2; **(F)** Nucleotides of VP3; **(G)** Amino acids of VP3.

A phylogenetic tree, represented by two large branches, was constructed based on the complete genome sequence of 44 reference strains and 12 isolates. All of the isolates were on the same large branch, which was closely related to the CIAV strains derived from Asia. After subdivision, the isolates became located to three different small branches. The four strains HN-3, HN-5, HN-7, HN-10, and HN-11 were on the same branch as the CIAV strains isolated from Shandong and Liaoning, and closely related to the classical attenuated strain C369 and CIAVV89-69. Another three strains HN-2, HN-4, and HN-6 appeared on the same branch as the HN1405 strain isolated from Shandong. HN-1, HN-8, HN-9, and HN-12 are on the same branch as the HN1504 strain. All of the CIAV strains isolated in Henan are far removed from the internationally prevalent vaccine strain Cux-1, and are not on the small branch with the Del-Ros vaccine strain (Figure 1A).

### Amino Acid Sequence Analysis of the VP1 and VP2 Proteins

Amino acid sequence analysis based on the VP1 sequence (450 aa) showed that the overall variation in VP1 protein from all of the isolates was 0–3.4%. The similarity in the VP1 amino acid sequence for the HN-1 and HN-12 strains reached 100% (Supplementary Figure 3). When comparing the VP1 protein amino acid evolutionary tree (Figure 1B), and the nucleotide sequence evolutionary tree (Figure 1C), we found that the VP1 nucleotide tree was roughly the same as the whole genome nucleotide evolutionary tree. When we compared the amino acid trees, we found HN-3, HN-4, and HN-7 were on the same branch as GD-1-12, while the HN-3 and HN-7 isolates were on the same branch as the HN-5 and HN-10 isolates. Finally, the HN-4 isolate was on the same branch as the HN-2 and HN-6 isolates. Therefore, it can be inferred that there may exist site mutations, or sequence recombinations in these strains at the amino acid level.

VP2 is the most conserved CIAV protein and there are no significant differences at the amino acid level between the isolates and the reference strain, and the co-efficient of variance was less than 2.3% Supplementary Figure 4. The VP2 nucleotide phylogenetic tree (Figure 1D) and amino acid phylogenetic tree (Figure 1E) displayed no obvious phylogenetic pattern. The VP3 protein from the isolates and reference strain, are more conservative at the amino acid level with a difference co-efficient of 0–1.7% (Supplementary Figure 5). The nucleotide phylogenetic tree (Figure 1F) and the amino acid phylogenetic tree (Figure 1G) of VP3 had no obvious phylogenetic pattern.

### Analysis of Major Amino Acid Sites in VP1, VP2, and VP3 Proteins

Previous studies have confirmed that the hypervariable region in the VP1 sequence is at amino acid position 13, in which amino acids 139 and 144 have an effect on the replication rate and infection efficiency of the virus in infected cells (21). When the 139 th and 144 th amino acids of the strain were glutamine (Q), the proliferation rate was significantly slower. In this study, the amino acid sites for the VP1 protein in the 12 isolates and previously isolated strains from Henan were all analyzed. Among these, HN-1, HN-8, HN-9, and HN-12 strains all carried Q139 and Q144, the same as those of the HN1504 strain. The other eight CIAV isolates (HN-2, HN-3, HN-4, HN-5, HN-6, HN-7, HN-10, and HN-11) carried the amino acid lysine (K) at position 139 and glutamate (E) at position 144. All the viruses isolated from Henan contained Q, at position 394, suggesting that the isolates from Henan had pathogenicity. HN-4, HN-6, and HN-8 had an A290P mutation, which has never been previously reported. In addition, there is a S287N mutation in the HN-4 strain and an S287T mutation in the HN-6 strain. However, whether this mutation influences its pathogenicity, needs further investigation (Table 2).

**Table 2:** Main amino acid positions in the VP1 protein.

**Virus strain**	**VP1 amino acid site**												
	**22**	**75**	**97**	**125**	**139**	**144**	**157**	**287**	**290**	**370**	**376**	**413**	**446**
Cux-1	H	V	M	I	K	D	V	A	A	S	L	A	T
Del-Ros	.	.	.	.	.	E	.	S	.	G	.	S	G
C369	.	.	.	L	.	E	.	S	.	G	I	S	S
GD-1-12	.	.	.	L	.	E	M	S	.	G	I	S	S
HN1405	.	.	.	L	.	E	.	S	.	A	.	.	S
HN1504	N	I	L	.	Q	Q	.	.	.	.	.	.	S
SMSC-IP60	.	.	.	.	.	E	M	S	.	G	.	.	.
SDLY08	N	.	.	.	.	E	.	S	.	G	.	S	.
HN-1	N	I	L	.	Q	Q	.	.	.	.	.	.	S
HN-2	.	.	L	L	.	E	M	S	.	A	.	.	S
HN-3	.	.	.	L	.	E	M	S	.	G	I	.	S
HN-4	.	.	L	L	.	E	M	N	P	G	I	S	S
HN-5	.	.	.	L	.	E	.	S	.	G	I	S	S
HN-6	.	.	L	L	.	E	M	T	P	A	.	.	S
HN-7	.	.	.	L	.	E	M	S	.	G	I	S	S
HN-8	N	I	L	.	Q	Q	.	.	P	G	I	.	S
HN-9	N	I	L	.	Q	Q	.	.	.	.	.	.	S
HN-10	.	.	.	L	.	E	.	S	.	G	I	S	S
HN-11	.	.	.	L	.	E	.	S	.	G	I	S	S
HN-12	N	I	L	.	Q	Q	.	.	.	.	.	.	S

The amino acid sites of the VP2 and VP3 proteins were relatively conserved with only a few mutated amino acid positions. In VP2, five alternative amino acid mutations were observed: G31E, A53V in the HN-1 isolate, S13R in HN-6 isolate, K102E in HN-7 isolate, and G24E in the HN-8 isolate. In VP3, P18S was observed in HN-1 isolates, and A54G was observed in HN-2 isolates (Table 3).

**Table 3:** Main amino acid positions in the VP2 and VP3 proteins.

**Virus strain**	**VP2 amino acid site**				**VP3 amino acid site**		
	**13**	**24**	**31**	**53**	**102**	**18**	**54**
Cux-1	S	G	G	A	K	P	A
Del-Ros	.	.	.	.	.	.	.
C369	.	.	.	.	.	.	.
GD-1-12	.	.	.	.	.	.	.
HN1405	.	.	.	.	.	.	.
HN1504	.	.	.	.	.	.	.
SMSC-IP60	.	.	.	.	.	.	.
SDLY08	.	.	.	.	.	.	.
HN-1	.	.	E	V	.	S	.
HN-2	.	.	.	.	.	.	G
HN-3	.	.	.	.	.	.	.
HN-4	.	.	.	.	.	.	.
HN-5	.	.	.	.	.	.	.
HN-6	R	.	.	.	.	.	.
HN-7	.	.		.	E	.	.
HN-8	.	E	.	.	.	.	.
HN-9	.	.	.	.	.	.	.
HN-10	.	.	.	.	.	.	.
HN-11	.	.	.	.	.	.	.
HN-12	.	.	.	.	.	.	.

### Recombination Sequence Analysis

**Figure 2:**
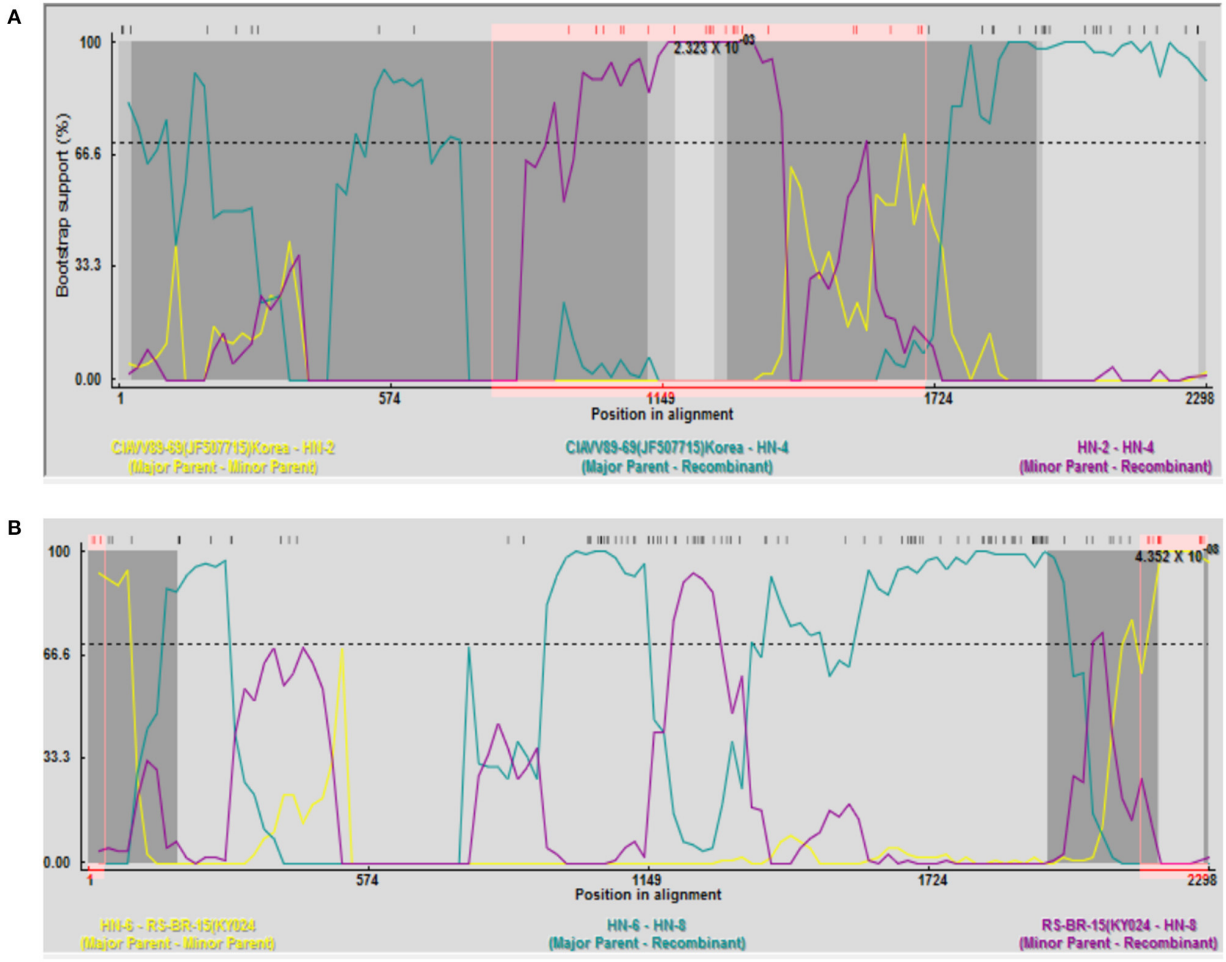
Recombination analysis of the **(A)** HN-4 strain; and **(B)** HN-8 strain. **(A)** Bootscan analysis of HN-4 strain and sequences of primary and secondary parents. Korean strain CIAVV89-69 was the main parent, and Henan strain HN-2 was the secondary parent, whose recombinant breakpoints were mapped to sites 787 and 1707. Bootscan analysis of HN-8 strain and sequence of primary and secondary parents. Henan strain HN-6 was the main parent, and Brazilian strain RS-BR-15 was the secondary parent, whose recombinant breakpoints were mapped to sites 36 and 2155.

Recombination analysis between all isolates and reference strains using RDP 4 software showed that recombination events occurred between different strains. Among them, HN-4 and HN-8 strains may be two potential recombinant strains. The isolate HN-4 may be a potential recombinant strain between the Korean isolate CIAVV89-69 and the Henan isolate HN-2. Bootscan analysis of the sequence of the HN-4 strain and its major and minor parental strains was conducted. The Korean strain CIAVV89-69 was found to be the major parental strain, while the Henan strain HN-2 represented the minor parental strain, and its recombination breakpoint was mapped to position 787 (initial breakpoint) and 1,707 (termination breakpoint) (Figure 2A).

Isolate HN-8 may represent a potential recombinant strain between the Brazilian isolate RS-BR-15 and the Henan isolate HN-6. Here, we used Bootscan analysis of the sequence of the HN-8 strain and its major and minor parental strains. We found that the Henan isolate HN-6 was the major parental strain, while the Brazilian isolate RS-BR-15 was the minor parental strain, and its recombination breakpoint was mapped to position 36 (initial breakpoint) and 2,155 (termination breakpoint) (Figure 2B).

### Pathogenesis of HN-4

**Figure 3:**
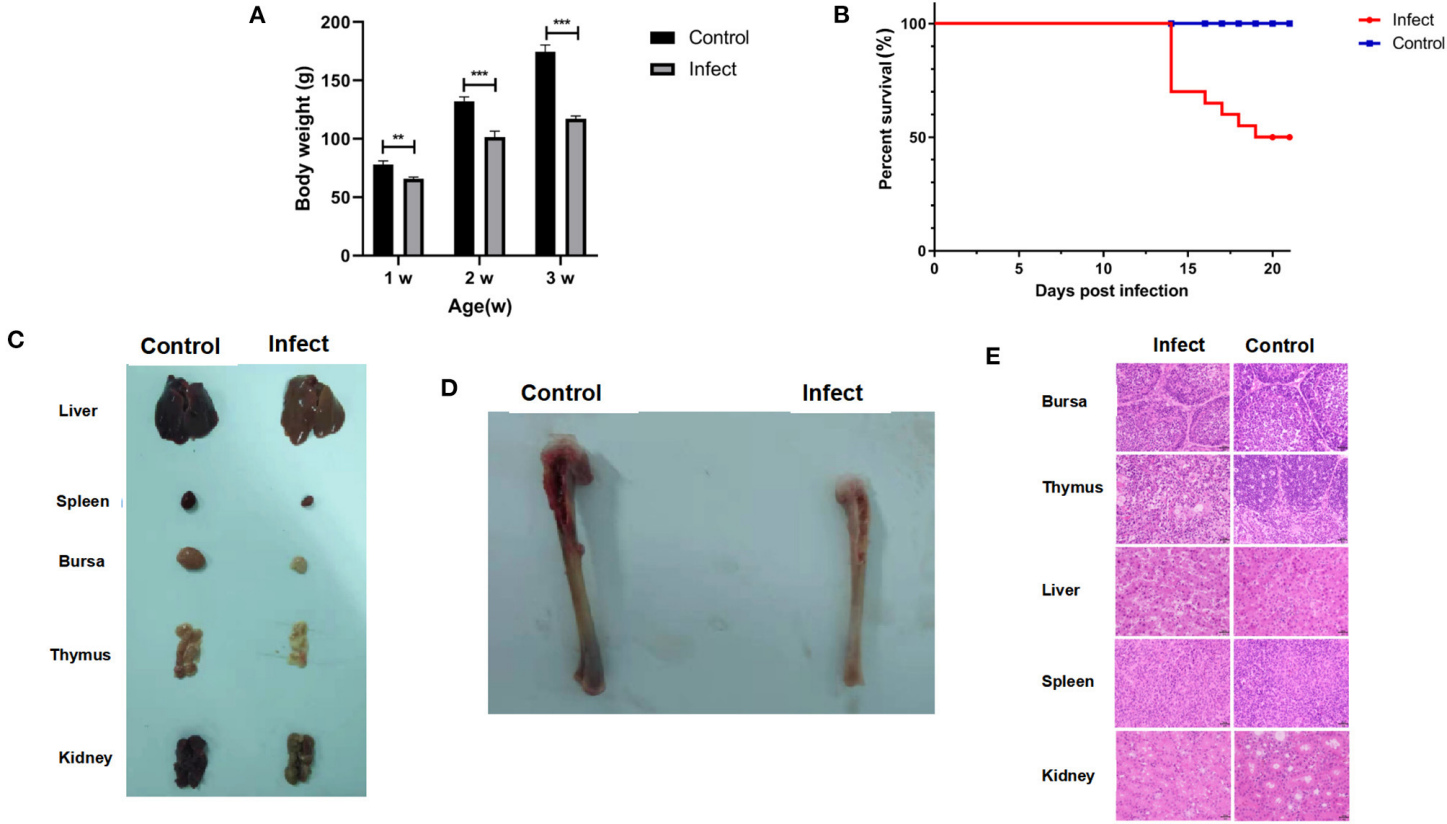
Pathogenic characterization of HN-4. **(A)** Body weight changes in chickens of different ages (weeks) after HN-4 infection; The statistical differences between groups were examined by student *t*-test. ** and *** represent *p* < 0.001. **(B)** Survival rate of chickens infected with HN-4. **(C)** Bone marrow changes in chickens infected with HN-4 after 16 days, Compared with the control group, there were no obvious pathological changes in liver and kidney, but obvious atrophy of spleen, bursa of Fabricius and thymus. **(D)** Pathological changes in various organs from chickens infected with HN-4 after 16 days, The tibia of the chicken infected with HN-4 is thin and fragile, and the bone marrow is yellow. **(E)** Histopathology in different tissues at day 16 post infection (H&E staining, original magnification ×400). The number of lymphocytes decreased significantly in bursa of Fabricius, thymus, spleen and kidney. Compared with the control group, the hepatocytes atrophied, the thymocytes edema and degeneration appeared “vacuole-like,” and the inclusion bodies in the virus nucleus could be seen. Among of the figures, infect means CIAV infection.

It has been found that CIAV could be isolated using MSB1 cells, but different strains have great differences in susceptibility infection (22). We found that only HN-4 strains and HN-6 strains could be propagated on MSB1 cells. Sequence analysis showed that only HN-4 has gene recombination. To examine the biological characteristics of the CIAV field isolates, we conducted analyses of their pathogenicity by infecting one-day-old SPF chickens with the HN-4 strain. As expected, there were no pathogenic symptoms in the control group. In the experimental group, however, no death was seen from day 1 to day 13, but chickens showed depression, were somnolent, displayed abnormal development and had a significantly lower body weight (*P* < 0.001) (Figure 3A). Chickens in the experiment group died continuously from 14 days post infection, with a total of 10 in 20 deaths seen up until the end of the experiment (21 days) (Figure 3B). At necropsy, no obvious pathological changes were seen in their livers and kidneys, but significant atrophy was seen in the spleen, bursa, and thymus, when compared to the control group (Figure 3C). The tibia of chickens infected with HN-4 was thin and fragile and had yellowish colored bone marrow (Figure 3D).

To further evaluate the pathogenicity of the HN-4 strain, histological lesions were examined on day 16 in the bursa, thymus, liver, spleen, and kidney. Our findings revealed that there was a significantly reduced number of lymphocytes in the Bursa, Thymus, spleen, and kidney. Whereas liver cells displayed metatropy when compared to the control group (Figure 3E). These results revealed that HN-4 is a pathogenic strain.

## Discussion

CIAV infection of its natural host, the chicken, results in a low mortality rate, however, CIAV infection does cause important symptoms such as a poor immune response and even immune failure in its host, resulting in invasion of secondary pathogens, which can then induce a huge economic loss to the poultry industry (23). Therefore, it is vital to perform epidemiological and pathological investigations to help control the spread of CIA. We performed a PCR survey of CIAV infection, which was carried out on 437 samples collected from September 2017 to January 2020, with a positive rate of 9.4% (95% CI: 6.8–12.5%), confirming the prevalence of CIA in the field, in the Henan province region of China.

In addition, the safety of a potential vaccine is closely related to the health of chickens (24). Here, we investigated the extent of CIAV contamination in the live attenuated non-CIAV vaccines sold at market in Henan Province. A molecular epidemiological survey was conducted on vaccine samples from 24 manufacturers, with a total of 120 attenuated non-CIAV vaccines. The results showed that the overall positive rate was 3.3% (95%CI: 0.9–8.3%), indicating that the necessity for biological product quality control. In the present study, CIAV was mainly tested in the Newcastle disease vaccine, bivalent live attenuated vaccine of Newcastle disease, Avian infectious bronchitis, and Fowlpox live vaccines. Possible CIAV contamination in other important avian diseases, also needs to be further investigated. Unfortunately, we did not isolate or identify live CIAV from these non-CIAV vaccines.

We did however, isolate and identify 12 CIAV strains from chicken samples, and then analyzed their genome sequences. The nucleotide variations among all the isolates ranged from 0.75 to 3.9%, indicating little difference between these strains and the reference strains. The high identity seen in the Henan isolates suggested that these strains are highly conserved. Phylogenetic results revealed that the 12 CIAV strains were in one large branch, most of which were Asian isolates. These isolates were further divided into three branches, where HN-3, HN-5, HN-7, HN-10, and HN-11 were all on the same branch as the Japanese classical attenuated strain C369 and the Korean attenuated strain CIAV 89-69 (25). Among them, HN-11 was on the same small branch as Guangdong isolate GD-1-12 (26). The three strains HN-2, HN-4, and HN-6 were on the same branch as the former Henan HN1405 strain, whereas the HN-1, HN-8, HN-9, and HN-12 strains were on the same branch as the former Henan HN1504 strain, indicating that these strains might be the dominant strains in Henan province. However, there were no similar branches seen for the international vaccine strains Cux-1 and Del-Ros (27). These results indicate that the Henan isolates may be epidemic strains, after a natural recombination and evolutionary event in Asia.

Of the three intact proteins found in the isolates, VP1 protein had the most amino acid site variations. Previous studies have confirmed that higher virulence strains are seen when position 394 in VP1 protein is Q, which represents the main genetic determinant of CIAV virulence (28). In this study, all isolates had Q at this position, indicating that the viral strains have strong virulence. HN-4, HN-6, and HN-8 have A 290 to P mutations at position 290, which has never been previously reported. However, VP2 and VP3 were relatively conserved, which may not affect their basic functions. These results suggested that the antigenic variation seen in VP1 may be important for virulence.

Recombination is the main source of mutation and the driving force for virus evolution (26). We tested the 12 isolated CIAV strains and found that there were genetic recombination events in the HN-4 and HN-8 strains. Recombination occurred both in the coding region and the non-coding region, suggesting a higher level of adaptability and epidemic transmission. Importantly, HN-4 infection induced 50% death and significant pathological symptoms after inoculation of one-day-old chickens in a 21-day long experiment. These results suggest that HN-4 might be a pathogenic epidemic strain induced by recombination.

In conclusion, our study has focused on the isolation, identification and analysis of different CIAV strains prevalent in Henan province, and found that there were specific amino acid site variations and gene recombinations. These recombination events may be responsible for the evolution of virulence in the epidemic strains, whose biological characteristics need further investigation.

## Data Availability Statement

The original contributions presented in the study are included in the article/Supplementary Material, further inquiries can be directed to the corresponding author.

## Ethics Statement

The animal study was reviewed and approved by Animal Ethics Committee of Henan Agricultural University.

## Author Contributions

X-WW, JF, and G-QZ conceived and designed the study. H-YL and J-JW performed the experiments. J-XJ, X-JZ, RW, XY, and LC analyzed the data. X-WW, JF, Y-FL, A-JS, and G-QZ wrote and revised the manuscript. All authors read and approved the final version.

## Funding

This work was supported by the National Natural Science Foundation of China (Nos. U21A20260 and U21A201577), the Starting Foundation for Outstanding Young Scientists of Henan Agricultural University (No. 30500690), the Advanced program of 2020 for returned overseas scholar (No. 30602136), the Key Scientific Research program of 2022 for Colleges and Universities in Henan Province (No. 22A230011), and Henan Fengyuan poultry Co., Ltd (Poultry farm disease control and eradication program).

## Conflict of Interest

The authors declare that the research was conducted in the absence of any commercial or financial relationships that could be construed as a potential conflict of interest.

## Publisher's Note

All claims expressed in this article are solely those of the authors and do not necessarily represent those of their affiliated organizations, or those of the publisher, the editors and the reviewers. Any product that may be evaluated in this article, or claim that may be made by its manufacturer, is not guaranteed or endorsed by the publisher.

## Abbreviations

CIA: Chicken infectious anemia
CIAV: Chicken infectious anemia virus
NDV: Newcastle disease virus
IB: Infectious bronchitis
FPV: Feline parvovirus
MS: Multiple sclerosis
ILTV: Infectious laryngotracheitis virus
ORF: Open Reading Frame
MSB1: Marek's disease lymphoblastoid cell
PBS: Phosphate-buffered saline
SPF: Specific pathogen free
PCR: Polymerase chain reaction
NCBI: National Center for Biotechnology Information
H&E: Hematoxylin-eosin.
