# Supplementary material for: Molecular Epidemiology and Pathogenic Characterization of Novel Chicken Infectious Anemia Viruses in Henan Province of China
Source: Front Vet Sci. 2022 Mar 28;9:871826. doi: 10.3389/fvets.2022.871826 (PMC8995968; doi:10.3389/fvets.2022.871826)
Supplement: Supplementary file 1 [file Data_Sheet_1.docx]

Supplementary Materials

**Supplementary Table 1** Reference CIAVs for Henan isolates sequence analysis

| Virus strain | Area | GenBank accession number |
| --- | --- | --- |
| 3-IP60 | Malaysia | AY040632.1 |
| 82-2 | Japan | D31965.1 |
| 98D02512 | USA | AF311892.2 |
| 704 | Australia | U65414.1 |
| C369 | Japan | AB046590.1 |
| CIAVV89-69 | Korea | JF507715.1 |
| clone34 | Germany | AJ297685.2 |
| Cux-1* | USA | M55918.1 |
| Del-Ros* | USA | AF313470.1 |
| L14767.1 | USA | L14767.1 |
| RS-BR-15 | Brazil | KY024579.1 |
| SMSC-1 | Malaysia | AF285882.1 |
| SMSC-IP60 | Malaysia | AF390102.1 |
| TR20 | Japan | AB027470.1 |
| AH4 | China | DQ124936.1 |
| CIAV Mouse | China | KU645525.1 |
| HN1405 | China | KU645520.1 |
| HN1504 | China | KU645512.1 |
| isolate 18 | China Taiwan | KJ728827.1 |
| JN1503 | China | KU641014.1 |
| LF4 | China | AY839944.2 |
| LN1402 | China | KU645511.1 |
| LY-1 | China | KX447636.1 |
| SD15 | China | KX811526.1 |
| SD22 | China | DQ141673.1 |
| SD1512 | China | KU645506.1 |
| SDLY08 | China | FJ172347.1 |
| TJBD40 | China | AY846844.1 |
| HA4 | China | DQ124934.1 |
| HLJ15108 | China | KY486137.1 |
| GD-K-12 | China | KF224935.1 |
| GD-1-12 | China | JX260426.1 |

Note: "*" represents vaccine virus.

# Supplementary Figures


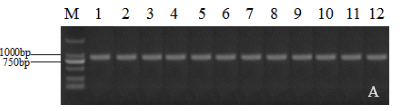

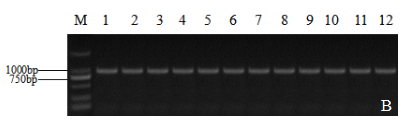

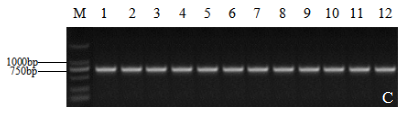


**Supplementary Figure 1** PCR analysis of CIAV isolates.


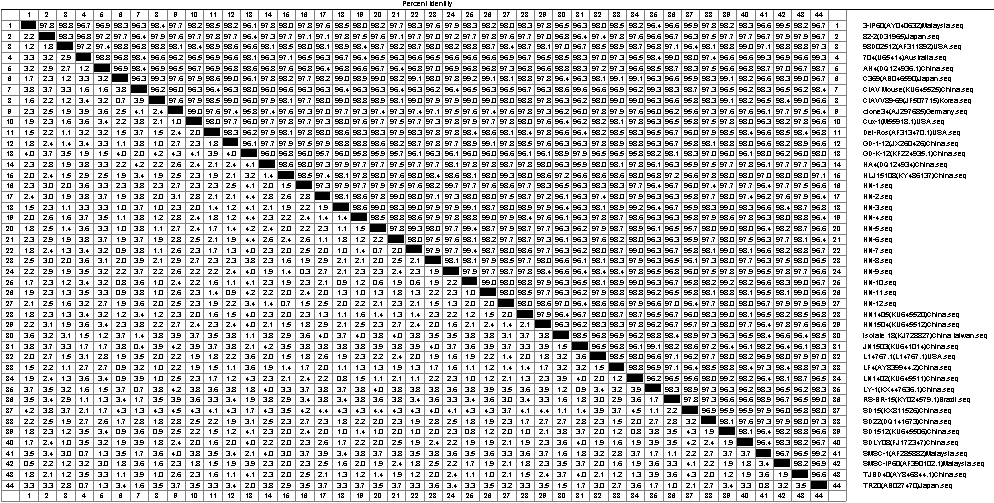


**Divergence**

**Supplementary Figure 2** Similarity analysis of the whole genome sequences of 12 CIAV isolates.


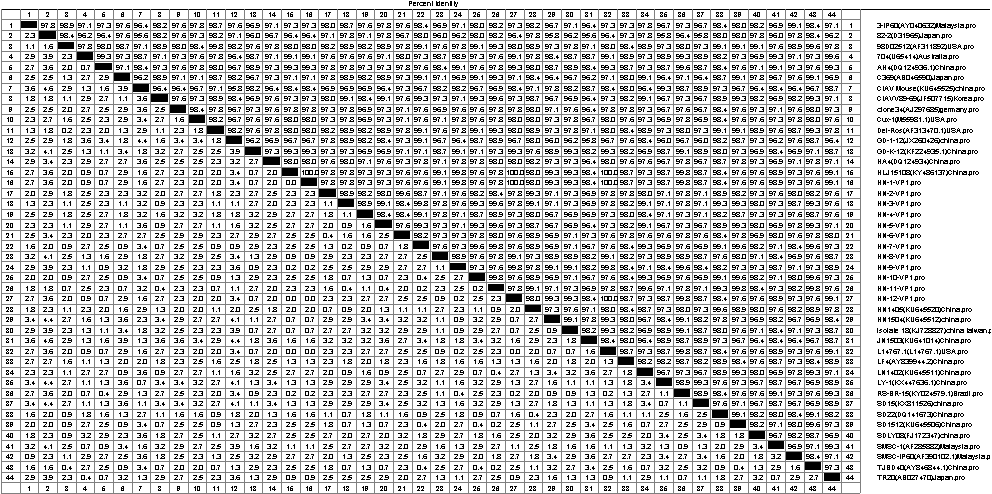


**Divergence**

**Supplementary Figure 3** The amino acid sequences similarity analysis of VP1.


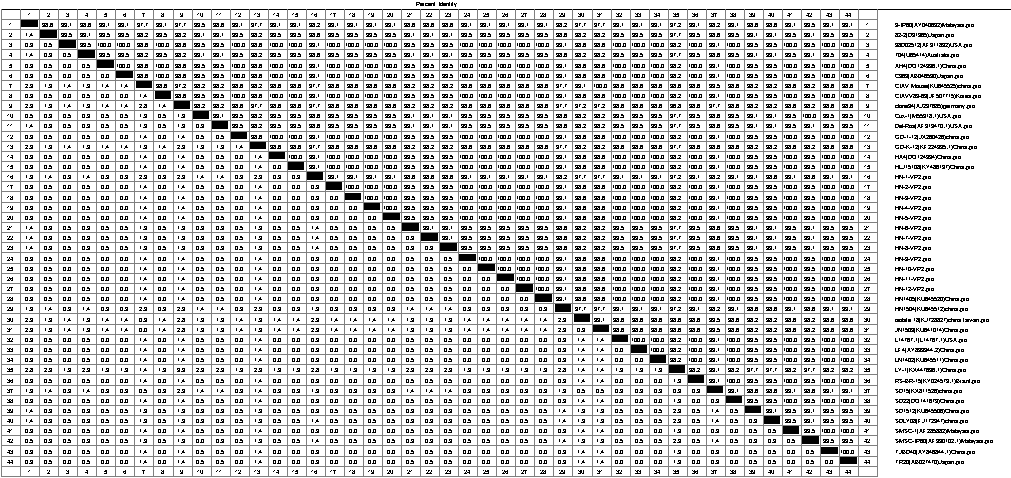


**Divergence**

**Supplementary Figure 4** The amino acid sequences similarity analysis of VP2.


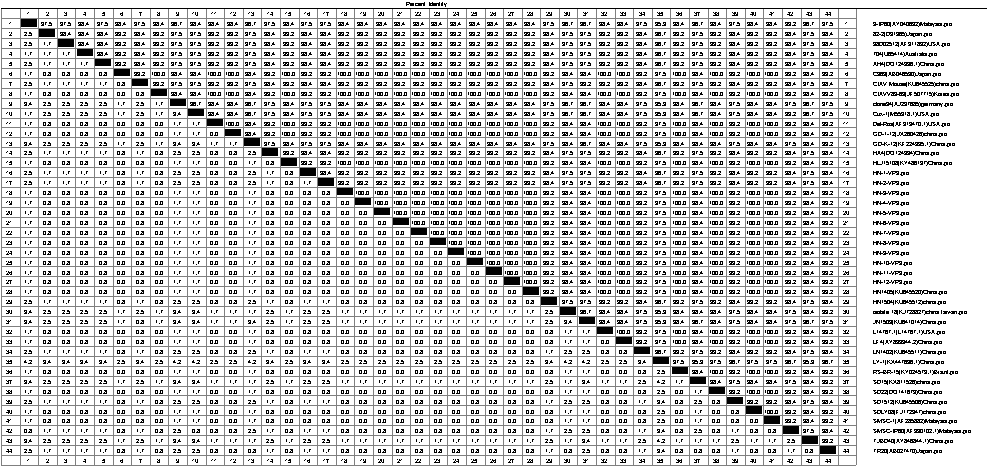


**Divergence**

**Supplementary Figure 5** The amino acid sequences similarity analysis of VP3.
